# Breast Cancer Risk in Rheumatoid Arthritis: An Update Meta-Analysis

**DOI:** 10.1155/2014/453012

**Published:** 2014-10-27

**Authors:** Guo Tian, Jia-Ning Liang, Zhuo-Yun Wang, Dian Zhou

**Affiliations:** ^1^School of Health Management, Anhui Medical University, 81 Meishan Road, Hefei, Anhui 230032, China; ^2^The First Affiliated Hospital of Anhui Medical University, 218 Jixi Road, Hefei, Anhui 230022, China; ^3^The Second Affiliated Hospital of Anhui Medical University, 678 Furong Road, Hefei, Anhui 230601, China

## Abstract

*Background*. The incidence of breast cancer in RA patients remains controversial. Thus we performed a meta-analysis to investigate the impact of RA on breast cancer. *Methods*. Published literature was available from PubMed, Embase, and Cochrane Library. Pooled standardized incidence rate (SIR) was computed by random-effect model analysis.* Results*. We identified 16 separate studies in the present study, in which the number of patients ranged from 458 to 84,475. We did not find the increased cancer risk in RA patients (SIR = 0.86, 95% CI = 0.72–1.02). However, subgroup analysis showed that breast cancer risk in RA patients was positively different in Caucasians (SIR = 0.82, 95% CI = 0.73–0.93) and non-Caucasians (SIR = 1.21, 95% CI = 1.19–1.23), respectively. In subgroup analysis by style, a reduced incidence was found in hospital-based case subjects (SIR = 0.82, 95% CI = 0.69–0.97). Similarly, subgroup analysis for adjusted factors indicated that in A3 (age and sex) and A4 (age, sex, and race/ethnicity) the risk was decreased (SIR = 0.87, 95% CI = 0.76–0.99; SIR = 0.63, 95% CI = 0.59–0.67).* Conclusions*. The meta-analysis revealed no increased breast cancer risk in RA patients. However, in the subgroup analysis, the risk of breast cancer is increased in non-Caucasians patients with RA while it decreased in Caucasian population, hospital-based case subjects, and A3 group. Such relationship may provide preference for risk of breast cancer in different population.

## 1. Introduction

Rheumatoid arthritis is a common type of autoimmune disease characterized by synovitis and joint destruction, which could lower the quality of life and life expectancy. With the improvement of treatment, the survival of RA patients has been increased. Recently, it has been reported that many comorbidities may arise during the RA disease course. Among these comorbidities, cancer is an attractive issue. Recently, many studies have analyzed the association between RA and breast cancer. Abnormal regulations of the host's immune surveillance are considered as cause of development of tumors [1]. Immune suppression might be responsible for the increased risk of many cancers, obvious after organ transplantation and immunodeficiency viral infections [2, 3]. The pathogenetic mechanisms of interaction between RA and cancer are still unknown. A previous meta-analysis by Smitten et al. observed a decrease in risk for breast cancer in RA patients many years ago without focusing on specific subgroup [4]. Recently, emerging studies have shown the possible risk of breast cancer in RA, but the incidence of breast cancer in this population remains controversial [5–7]. This update review aims to compare the incidence of breast cancer in patients with RA versus the general population through observational studies. We hope that this could offer an actual view of clinically estimating the risk.

## 2. Material and Methods

### 2.1. Search Strategy

This meta-analysis was conducted with reference to the following standard guidelines [8]. Relevant publications were identified through a systematic literature search using the keywords “RA,” “Rheumatoid Arthritis,” “breast,” “tumor,” “tumour∗,” “carcinoma∗,” “oncology∗,” “neoplas∗,” “cancer,” “malignanc∗,” “standardized incidence rate,” and “SIR” in PubMed, Embase, and Cochrane Library. Other literatures were available from cross-references within both original and review articles. We only collected data from the full-published paper, excluding any meeting or conference abstract. No language or race restrictions were applied.

### 2.2. Inclusion Criteria

A recruited study had to meet the following criteria: (1) it was a cohort study; (2) sample size was not less than 100 patients; (3) the study offered necessary information like standardized incidence rate (SIR) and its 95% confidence interval (CI) of breast cancer in RA patients.

### 2.3. Exclusion Criteria

The exclusion criteria were as follows: (1) conference abstracts, case reports, and review articles; (2) not cohort studies; (3) not control subjects; (4) duplicate reports or unclear data description. If the same cohort study appeared in other publications, only the latest article was selected into our study.

### 2.4. Data Extraction

Information was carefully extracted from these recruited publications independently by two of the authors (by Guo Tian and Jia-Ning Liang), including author, year of publication, country, calendar period, total number of RA patients, person-year of follow-up, sources of patients, observed/expected, and standardized incidence rate (SIR) with its 95% CI. If original necessary data were unavailable in relevant articles, a request was sent to the author for additional data.

### 2.5. Literature Quality Assessment

An independent literature search was performed (by Guo Tian and Jia-Ning Liang) with the same method. The contents of abstracts were checked independently by two investigators (Guo Tian and Zhuo-Yun Wang) to decide whether they were available for inclusion. References in the studies were reviewed (by Guo Tian and Zhuo-Yun Wang) to identify additional studies. When discrepancies occurred, a third investigator (Dian Zhou) made further assessment and a final decision was made by the majority of the votes. The Newcastle-Ottawa quality assessment scale was implemented to assess the quality of the methodology in the included studies. This scale comprising eight questions with nine possible points was reviewed by a star system according to the selection populations, comparability of groups, and outcome. The qualities of the recruited studies were evaluated and examined by two reviewers (Guo Tian and Jia-Ning Liang), respectively.

### 2.6. Statistical Analysis

In the meta-analysis, we used SIR with its 95% CI to combine these pieces of data. Heterogeneity assumption in studies was checked with the *Q* statistic [9]. Meanwhile, we assessed the effect of heterogeneity in these studies by the following method: *I* ^2^ = 100% × (*Q* − df)/*Q* [10]. A significant *Q* statistic (*P* < 0.10) suggested heterogeneity across studies, and then the result of the random-effect model was selected. If not, the result of the fixed effect model was selected. Additionally, publication bias was investigated with the funnel plot and Egger's linear regression test [11]. All the analyses were performed using the software Stata 12.0 version (StataCorp LP, College Station, TX, USA).

## 3. Results

### 3.1. Characteristics of Eligible Studies

**Figure 1:**
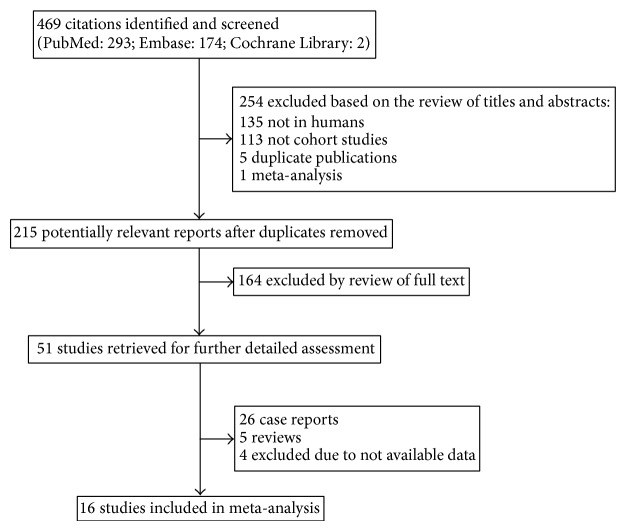


Characteristics of studies eligible for the current meta-analysis appeared in Table 1. The study selection process is shown in Figure 1, and four hundred fifty-three of 469 papers were excluded (135 not in human; 113 not cohort studies; 5 duplicate publications; 1 meta-analysis; 26 case reports; 5 reviews; 4 not available data). A total of final 16 articles met our inclusion criteria [5–7, 12–24]. These cohort studies ranged from 458 to 84,475 patients and had mean follow-up times from 3.7 to 10 years.

### 3.2. Study Quality

With regard to cohort studies, 83% were of high quality (NOS score > 6), with an average NOS score of 6.2. The quality ratings of each study according to NOS criteria are listed in Table 2. Given the comparability, the quality was relatively low because many studies did not report on the control for RA, an essential potential confounder. Additionally, four studies definitely fulfilled the American College of Rheumatology classification criteria for diagnosis of RA, while in the other studies the diagnostic criteria for RA were unknown.

### 3.3. Meta-Analysis Results

#### 3.3.1. Analysis in Patients with RA

**Figure 2:**
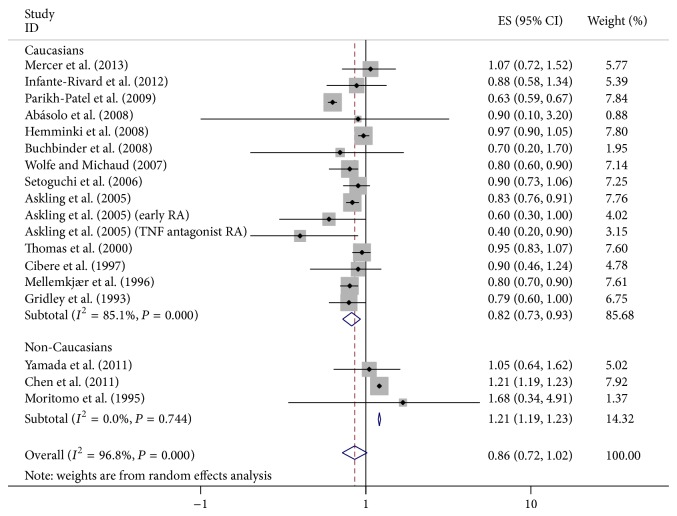


**Figure 3:**
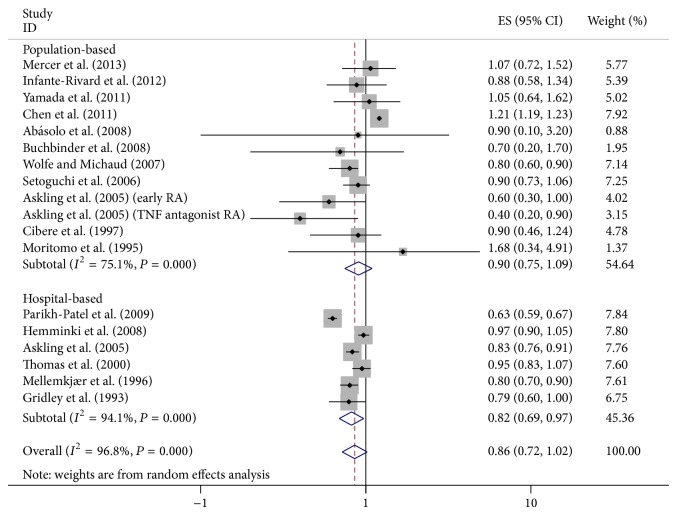


**Figure 4:**
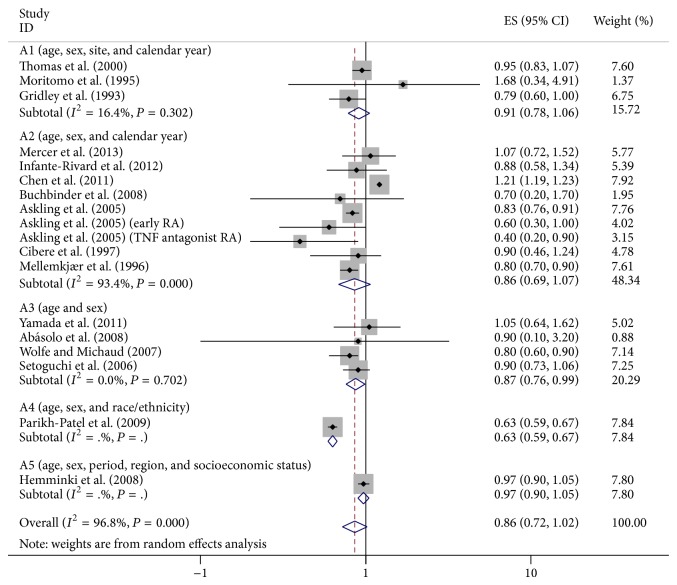


The *Q*-test of heterogeneity was not significant and then the original SIRs were pooled by means of the random-effect models (*P* < 0.001, *I* ^2^ = 96.8%). We did not detect the increased cancer risk in RA patients (SIR = 0.86, 95% CI = 0.72–1.02). Subgroup analysis was performed by ethnics. It showed that breast cancer risk in RA patients was positively different in Caucasians and non-Caucasians (Figure 2). Likewise, in subgroup analysis by style, a reduced incidence was found in hospital-based case subjects but not in population-based subjects (Figure 3). And in subgroup analysis for adjusted factors, the risk was decreased in A3 and A4 groups (Figure 4).

#### 3.3.2. Sensitivity Analysis and Publication Bias

We conducted a sensitivity analysis in each group to assess the stability of this meta-analysis. When any single study was removed, the relevant pooled SIRs were not radically changed. It well suggested the stability of the meta-analysis. Funnel plot asymmetry was evaluated by the method of Egger's linear regression test. The result showed that there was a significant publication bias in the total population while not existing in Caucasians and non-Caucasians, respectively (*t* = −2.47, *P* = 0.025; *t* = 0.49, *P* = 0.633; and *t* = −0.1, *P* = 0.939). And publication bias was observed in population-based group but not in hospital-based group (*t* = −3.59, *P* = 0.005 and *t* = 0.76, *P* = 0.49). Also, A1 (age, sex, site, and calendar year) and A3 revealed no publication bias except A2 group (*t* = 0.14, *P* = 0.911; *t* = 0.56, *P* = 0.629; and *t* = −2.65, *P* = 0.033).

## 4. Discussion

Breast cancer is one of the worst death-related cancers for women in the world [25]. Reduced risk of breast cancer was observed in Caucasians patients with RA. These results are consistent with previous reports from Western countries in RA patients [6, 7, 12–22, 24]. It has been reported that the epidemiology of malignancy in Japan differed from that in Western countries. In Japanese population, breast cancer became the worst type of tumor followed by uterus and stomach cancers in women [6].

Currently, the RA patients in Taiwan are mainly treated by simultaneously using kinds of immunomodulatory drugs, such as prednisolone, NSAIDs, methotrexate, and biologic agents. In addition, antirheumatic drugs like azathioprine, hydroxychloroquine, cyclophosphamide, and mycophenolate mofetil are applied at times. Immunosuppressive therapy may increase the cancer risk in the population [7]. In particular, long-term continuous administration of them could add a great risk of developing opportunistic infections and cancer [26]. However, in these studies, the detailed doses were unclear. Thus, the discrepancy of doses of medication in Caucasians and non-Caucasians RA patients could result in different breast cancer incidence. It needs further studies to explore.

Breast density and BMI are needed to understand the possible implications of the increased breast cancer risk among RA patients from four case-control studies [27]. It is reported that diverse patterns of breast density by ethnicity are consistent with ethnic differences in breast cancer risk. With regard to the percentage of breast density, Asian women have higher breast density but Pacific women have lower breast density. Breast density may be a potentially important factor to New Zealand's well-known inequalities in breast cancer incidence. Asian women in American studies have been detected to have both lower [28] and higher breast densities in contrast with white women. Nevertheless, these results are possibly affected by the method of measuring breast density and confounding by various mean age at menopause [29]. It indicated that percent density could be not a marker of ethnic differences due to different breast size and significant variation by ethnicity [28].

It has been attractive for several decades about resemblance between the pathologies of autoimmune diseases and cancer. Though cancer study traditionally focuses on the tumor cells, host/tumor cell interactions in the tumor microenvironment are gradually regarded as important elements of tumor progression [30]. Their interactions both in the tumor and in the adjacent stromal cells could lead to the expression of stromal factors, including growth factors, chemokines, cytokines, proteases, and vascular-stimulating factors [31]. Carcinoma-associated fibroblasts (CAFs) appear in inflammatory environments, like RA, where they may be main components of the proliferating pannus and lead to angiogenesis and matrix remodeling [31]. In breast cancer, CAFs also become important cellular components of the tumor microenvironment [30]. CAFs can be regarded as a heterogeneous population [32]. In spite of their essential role in tumor progression, the features of CAFs need to be further clarified. In addition, elevated levels of IL-6 have been linked to poor prognosis in breast cancer patients and IL-6 plays vital roles not only in tumorigenesis, but also in inflammatory diseases including RA [33, 34]. They may partly explain the disease pathology of breast cancer in RA.

Recently, emerging studies have suggested that genetic polymorphism may play an important role both in the RA and in breast cancer. Li et al. [35] conducted a meta-analysis in Asian and non-Asian patients and found that miRNA-210 may be a better tumor predictor in Asian breast cancer patients. Cyclooxygenase two (COX-2) is a vital enzyme metabolizing arachidonic acid. Polymorphism −765 G/C in COX-2-encoding gene promoter is concerned with development of breast cancer and RA [36]. Glinskii et al. [37] found rs2670660 allele-specific gene expression signatures which seem to be suitable for investigating the activated states of innate immunity pathways from human disorders like RA and breast cancer. Nuclear factor-*κ*B (NF-*κ*B) regulates many genes for immune response, cell adhesion, differentiation, and proliferation. NF-*κ*B dysregulation may be associated with both inflammatory diseases and immune deficiencies like RA and several cancers including breast cancer [38]. In addition, the correlation of some estrogen receptor (ER)*α* genotypes with breast cancer [39] and some autoimmune diseases including RA [40] has been previously reported. In the different populations, various life styles and environmental factors result in different gene-environment interactions and thus might explain different cancer susceptibilities [41].

In subgroup analysis by style, although a reduced incidence was found in hospital-based case subjects in 6 cohort studies, the use of them might have led to selection bias because those suffering from mild RA would have made them more inclined to be away from hospitals. Nevertheless, this selection bias was minimized in the remaining population-based cohort studies.

Within stratified analysis for adjusted factors, it seems that the decreased age- and sex-specific incidence may be potential factors to decrease the risk. For breast cancer with the increased SIR, an early age (especially less than 50 years) of RA diagnosis was a risk factor [15]. Even another study showed that this SIR was 2.19 in females with age < 40. However, the risk tended to be reduced with aging [13]. Those elder RA patients might have better overall health than the general population. But younger RA patients with higher risks could be weaker in the general population. They may face more risk factors for breast cancer.

The present meta-analysis has several limitations. First, the sample size of non-Caucasians was relatively small, result of which may be partly biased, and thus the analysis may have insufficient statistical power to obtain a more real SIR and its 95% CI. Second, publication bias among included studies was identified while not detected when stratified by ethnicities. It is possible that some studies were not included in this analysis or some unpublished studies with null results were not found. Third, a more precise analysis could be conducted, if individual data including cultural factors, age, menopausal status, smoking, and other environmental factors may account for the ethnic difference between them. Fourth, a relatively high level of heterogeneity showed the instability of the result, which could come from the data sources.

In conclusion, the present study indicates that the breast cancer risk is not increased in overall RA patients. Nevertheless, increased risk of breast cancer is observed in non-Caucasians patients with RA while decreased risk is detected in Caucasian population, hospital-based case subjects, and A3 group. Although breast cancer risk prediction remains imperfect, this might provide preference for risk of breast cancer in different population. However, potential mechanism between RA and breast cancer risk is still unclear. Therefore, the conclusion should be interpreted with caution and more large-scale studies are warranted to confirm the results in the future.

## Figures and Tables

**Table 1:** Summary of studies of RA and breast cancer incidence.

Study	Calendar period	*N* total	Sources of RA patients	Style	Mean/median follow-up (year)	Breast cancer (observed/expected)	Adjusted factors for SIR	SIR (95% CI)
Mercer et al., (2013) [5]	2002–2009	3,771	The BSRBR (British Society for Rheumatology Biologics Register) Control Centre Consortium	Population-based	3.7	30/28.16	Age, sex, and calendar year	1.07 (0.72–1.52)
Infante-Rivard et al., (2012) [12]	1976–2001	1,437	Quebec Tumuor Registry, Quebec Institute of Statistics and Health, Canada	Population-based	5	22/25.01	Age, sex, and calendar year	0.88 (0.58–1.34)
Yamada et al., (2011) [6]	2001–2005	7,566	IORRA (Institute of Rheumatology, Rheumatoid Arthritis formally known as J-ARAMIS)	Population-based	NA	20	Age and sex	1.05 (0.64–1.62)
Chen et al., (2011) [7]	1996–2007	23,644	The National Health Research Institute of Taiwan	Population-based	5.90 ± 2.87	123/101.44	Age, sex, and calendar year	1.21 (1.19–1.23)
Parikh-Patel et al., (2009) [13]	1991–2002	84,475	California's Office of Statewide Planning and Health Development	Hospital-based	4.8	842/1343.5	Age, sex, and race/ethnicity	0.63 (0.59–0.67)
Abásolo et al., (2008) [14]	1999–2005	789	The EMECAR cohort (Estudio de la Morbilidad y Expresion Clinica de la Artritis Reumatoide)	Population-based	3.95	2	Age and sex	0.90 (0.10–3.20)
Hemminki et al., (2008) [15]	1980–2004	42,262	The Swedish Hospital Discharge Register	Hospital-based	NA	642	Age, sex, period, region, and socioeconomic status	0.97 (0.90–1.05)
Buchbinder et al., (2008) [16]	1986–1998	458	Victorian State Cancer Registry	Population-based	9.3	4/6.1	Age, sex, and calendar year	0.70 (0.20–1.70)
Wolfe and Michaud (2007) [17]	1998–2005	13,869	The US National Data Bank for Rheumatic Diseases	Population-based	4.1	102	Age and sex	0.80 (0.60–0.90)
Setoguchi et al., (2006) [18]	NA	29,422	Health care utilization databases from US and Canada	Population-based	NA	112/126.6	Age and sex	0.90 (0.73–1.06)
Askling et al., (2005) [19]	1990–2003	53,067	The Swedish Inpatient Register	Hospital-based	5.6	471	Age, sex, and calendar year	0.83 (0.76–0.91)
Askling et al., (2005) [19] (Early RA)	1995–2003	3,703	The Early Arthritis Register	Population-based	3.6	13	Age, sex, and calendar year	0.60 (0.30–1.00)
Askling et al., (2005) [19] (TNF antagonist RA)	1999–2003	4,160	Swedish structured postmarketing surveillance program	Population-based	2.3	8	Age, sex, and calendar year	0.40 (0.20–0.90)
Thomas et al., (2000) [20]	1981–1996	26,623	Scottish Cancer Registry	Hospital-based	5.3	249	Age, sex, site, and calendar year	0.95 (0.83–1.07)
Cibere et al., (1997) [21]	1966–1974	862	Rheumatic Disease Unit	Population-based	17.4	18/22.8	Age, sex, and calendar year	0.90 (0.46–1.24)
Mellemkjær et al., (1996) [22]	1977–1987	20,699	Danish Hospital Discharge Register	Hospital-based	7	186/229.8	Age, sex, and calendar year	0.80 (0.70–0.90)
Moritomo et al., (1995) [23]	1980–1989	655	Center for Adult Diseases, Osaka, Japan	Population-based	10	3/1.79	Age, sex, site, and calendar year	1.68 (0.34–4.91)
Gridley et al., (1993) [24]	1965–1983	11,683	Swedish Hospital Inpatient Register	Hospital-based	8.6	106	Age, sex, site, and calendar year	0.79 (0.60–1.00)

N: the number of RA patients.

NA: not available.

**Table 2:** Quality assessment of included studies.

Study	Whether the American College of Rheumatology (ACR) or not	Selection				Comparability	Outcome			Total awarded stars
		1 Representativeness of the exposed cohort	2 Selection of the nonexposed cohort	3 Ascertainment of exposure	4 Demonstration that outcome of interest was not present at start of study	1 Comparability of cohorts on the basis of the design or analysis	1 Assessment of outcome	2 Was follow-up long enough for outcomes to occur?	3 Adequacy of follow-up of cohorts (<20%)	
Mercer et al., (2013) [5]	NA	★	★	★	NA	★	★	★	NA	6
Infante-Rivard et al., (2012) [12]	NA	NA	★	★	NA	★	★	★	NA	6
Yamada et al., (2011) [6]	Y	★	NA	★	NA	★	★	★	NA	7
Chen et al., (2011) [7]	Y	★	★	★	★	★	★	★	NA	7
Parikh-Patel et al., (2009) [13]	NA	NA	★	★	★	★	★	★	NA	6
Abásolo et al., (2008) [14]	Y	★	★	★	NA	★	★	★	★	7
Hemminki et al., (2008) [15]	NA	★	★	★	★	★	★	★	NA	6
Buchbinder et al., (2008) [16]	Y	★	★	★	NA	★	★	★	NA	6
Wolfe and Michaud (2007) [17]	NA	★	★	★	NA	★	★	NA	NA	5
Setoguchi et al., (2006) [18]	NA	★	★	★	NA	★	★	★	NA	6
Askling et al., (2005) [19]	NA	★	★	★	NA	★	★	★	NA	7
Thomas et al., (2000) [20]	NA	★	★	★	NA	★	★	★	★	7
Cibere et al., (1997) [21]	NA	★	★	★	NA	★	★	★	NA	5
Mellemkjær et al., (1996) [22]	NA	★	★	★	NA	★	★	★	★	7
Moritomo et al., (1995) [23]	NA	★	★	★	NA	★	★	★	NA	6
Gridley et al., (1993) [24]	NA	★	★	★	★	★	★	★	NA	6

NA: not available.

Y: yes.

★: available.
